# Streamlined molecular farming of plant virus therapeutics for space flight and other low-resource environments

**DOI:** 10.1038/s44383-026-00030-y

**Published:** 2026-06-05

**Authors:** Patrick Opdensteinen, Kyle Lewin, Anshal Jain, Andrew Copeland, Jonathan Copeland, Maziar Ghazinejad, Nicole F. Steinmetz

**Affiliations:** 1 Aiiso Yufeng Li Family Department of Chemical and Nano Engineering, University of California San Diego, La Jolla, CA USA; 2 Center for Nano-ImmunoEngineering, University of California San Diego, La Jolla, CA USA; 3 Shu and K.C. Chien and Peter Farrell Collaboratory, University of California San Diego, La Jolla, CA USA; 4 Department of Electrical and Computer Engineering, University of California San Diego, La Jolla, CA USA; 5 Department of Mechanical and Aerospace Engineering, University of California San Diego, La Jolla, CA USA; 6 Department of Bioengineering, University of California San Diego, La Jolla, CA USA; 7 Department of Radiology, University of California San Diego, La Jolla, CA USA; 8 Institute for Materials Discovery and Design, University of California San Diego, La Jolla, CA USA; 9 Moores Cancer Center, University of California San Diego, La Jolla, CA USA; 10 Center for Engineering in Cancer, Institute of Engineering in Medicine, University of California San Diego, La Jolla, CA USA

**Keywords:** Biotechnology, Plant sciences

## Abstract

The resupply of pharmaceuticals during long-term space missions is challenging due to long travel distances and the reduced shelf-life of pharmaceuticals under extraterrestrial conditions. On-demand pharmaceutical production using plants could address this limitation. Plants are already cultivated in space, but the production of pharmaceuticals in plants is hampered by traditionally complex purification processes. Here, we describe a simplified production and purification strategy for cowpea mosaic virus (CPMV), a plant virus-based therapeutic candidate with strong immunomodulatory properties suitable for cancer therapy and vaccine development. By combining vacuum infiltration and centrifugation, intact CPMV particles were recovered from the apoplast without damaging the plant tissue, eliminating the need for tissue disruption. Impurities in apoplast eluates were efficiently removed by ultrafiltration/diafiltration, exploiting the size difference between CPMV and contaminants. The process was scalable when applied to more than 50 plants. We assessed the robustness of this production process under simulated space conditions, including microgravity, temperature shifts, and exposure to reactive oxygen species (ROS). Microgravity altered plant morphology, whereas temperature changes and ROS stress affected CPMV yields in a time-dependent manner. Beyond applications in space, these findings enable terrestrial strategies for plant molecular farming in low-resource environments.

## Introduction

A major challenge for long-term space missions is the need to resupply astronauts with pharmaceuticals despite the extreme travel distances, such as the 200 days of interplanetary travel to Mars^1^. The reduced shelf-life of pharmaceuticals in space compared to terrestrial conditions aggravates the resupply problem^2^. Even when assuming a terrestrial shelf-life, more than half of the medications used on the International Space Station (ISS) were found to expire within 36 months, making them barely sufficient for the completion of a 3-year Mars mission^3^. Potential consequences include the higher risk of therapeutic failure or toxicity^4–6^. On the ISS, this problem is addressed by replacing medication lots that are within 6 months of expiration, but this strategy is not feasible for long-term space missions^2^. The limited storability of pharmaceuticals in space also makes it difficult to stockpile them for the treatment of long-term conditions, which would be necessary during missions that span months or even years^7^. Life-saving biologics, such as antibodies, have not yet been tested on space flights^8^. However, this category of drugs is even more susceptible to degradation than small molecules^8,9^. The on-demand synthesis of biologics is currently not considered viable due to the molecular complexity and low yields of the products^10,11^. As mission durations and distances from Earth increase, on-demand biomanufacturing capacity will become increasingly important on spacecraft^8,12,13^.

Plant molecular farming, defined as the use of plants to produce biologics^14^, is now sufficiently mature as a technology to be considered as a platform for the on-demand production of biologics during space missions^8^. Plants offer significant potential for the manufacture of pharmaceuticals in space because they can produce small-molecule compounds and proteins in non-sterile environments^15^. In contrast, mammalian cells, yeast and bacteria require sterile bioreactors and thus additional equipment^8^. Unlike bacteria and mammalian cells, plants do not support the replication of human viruses^16^, nor do they require animal-derived components such as serum^17^, and thus offer a better safety profile. Additionally, plants can be brought into space as seeds, thus minimizing transport costs, which can exceed $1400 kg^−1^ even with commercial launch systems^18^. Transient production strategies (i.e., gene expression with an early maximum and a subsequent decline) allow the expression of recombinant proteins within hours^19^. Beyond their ability to produce pharmaceuticals, plants can also support deep-space missions by recycling water and air. Plants also have a positive influence on astronaut psychology^20^, which has been recognized as an important factor for astronaut wellbeing and ultimately for the success of space missions^21^. Such synergistic effects are desirable when considering that deep-space missions represent a severely resource-limited environment.

A major drawback that limits the broader utilization of plant molecular farming is the need to remove large quantities of soluble and insoluble impurities that are released during extraction^22^. This is even more problematic on spacecraft, where resources for purification are severely limited^8^. Extraction is typically achieved by blending the plant tissue, which creates large amounts of waste biomass (unused weight). Non-destructive extraction protocols would preserve the plants, thus minimizing waste and allowing plants to continue recycling water and air, as well as allowing multiple harvesting and purification cycles. This could be achieved by secreting products to the apoplast, the cell compartment immediately under the cell wall, from which the protein molecules can be eluted using a non-disruptive technique known infiltration-centrifugation^23,24^. Daily infiltration-centrifugation cycles are feasible for at least 6 days^25^, improving pharmaceutical protein yields per weight of leaf tissue^24^. Further improvements can be expected by engineering devices that apply the infiltration-centrifugation technique to entire plants. This approach has been established for antibodies, enzymes and growth factors under terrestrial conditions^26–28^, but has yet to be evaluated for space travel or for the purification of plant viruses and virus-like particles (VLPs).

Here, we investigated whether cowpea mosaic virus (CPMV), a plant virus with promising therapeutic properties, can be produced and purified by infiltration-centrifugation under the conditions found in space (microgravity and oxidative stress). Plant virus nanoparticles (VNPs) based on CPMV are ideal as a model to establish plant molecular farming in space because VNPs can be used as a platform technology to address astronaut health issues caused by the unique environment of space. One of the most severe side effects of spaceflight is the dysregulation of the immune system^7^. We and others have shown that plant viruses as well as engineered VNPs have remarkable immunomodulatory properties^29^, allowing these biologics to be repurposed as immunotherapies or vaccines targeting cancer^30,31^, chronic disorders^32^ including cardiovascular disease^33,34^, as well as infectious diseases^35–37^. We chose CPMV because the native virus has demonstrated efficacy against multiple types of cancer in mouse tumor models and canine cancer patients, eliminating tumors as well as distant metastases, and preventing recurrence^38–40^ following intra-tumoral^38^ or systemic delivery^41,42^ via the activation of pattern recognition receptors^43^. Despite its immunomodulatory properties, CPMV does not infect or replicate in mammalian cells^44^, and therefore does not pose a threat to humans. We assessed the resilience and scalability of the new production process under simulated space conditions, testing the effects of microgravity, temperature, and reactive oxygen species (ROS) on plant growth and CPMV yields.

## Methods

### Plant cultivation

*Nicotiana benthamiana* (in-house seeds) and black-eyed pea (*Vigna unguiculata* no. 5, Morgan County Seeds) plants were seeded in 7.5 × 7.5 × 6.5 cm (*L* × *W* × *H*) stone wool blocks (Hort Americas) and grown in an A1000 chamber (Conviron) at 25/22 °C (day/night cycle), 60% relative humidity and ~100,000 lux (16-h photoperiod) as previously described^45^. Stone wool blocks were placed in RooTrimmer 1020 Trays (Greenhouse Megastore) filled to a constant 2.5-cm depth with tap water. Black-eyed peas received one dose of fertilizer (JR Peters, #77860) at a concentration of 0.5 g/L after seeding. *N. benthamiana* plants received the same fertilizer at the same concentration after seeding and then every 2 weeks of cultivation. *N. benthamiana* plants were grown for 4 weeks before they were used for experiments, whereas black-eyed pea plants were grown for 1 week.

### Simulation of microgravity

Microgravity conditions were simulated using a custom-built random positioning machine (RPM). The device consisted of two aluminum frames that could be rotated independently using two RMD-L-7025 electric motors (MyActuator). An Arduino Uno and Raspberry Pi (RPI4) with a custom Python script were used to rotate biological samples in three dimensions, randomizing the influence of gravity. An accelerometer (Adafruit LSM6DS3TRC) was used to monitor the acceleration of the samples, which were mounted in the center of the device to minimize residual acceleration. The device was operated at 7 rpm for all experiments.

For plant growth experiments, the RPM was housed in a walk-in growth chamber (MTS144, Conviron) to control temperature, humidity and photoperiod. Default conditions were 25/22 °C (day/night cycle), 60% relative humidity and ~100,000 lux (16 h photo period) as described above^45^. The light source was positioned outside of the RPM, thus preventing plants from orienting themselves based on the angle of light incidence. For all experiments, static ground controls (1 × *g*) were grown under the same conditions.

Plants were infected with CPMV by the mechanical inoculation of primary leaves after 2 days of exposure to simulated microgravity as previously described^45^. After infection, plants were cultivated under simulated gravity conditions for 2 weeks before analyzing the effects on morphology, chlorophyll content and CPMV accumulation.

### Simulation of oxidative stress

The effect of oxidative stress on plant growth and CPMV yields was tested by applying hydrogen peroxide^46^. Plants were sprayed with 100 µM hydrogen peroxide in deionized water three times per day (~1.5 mL per plant)^47^, starting 2 days before infecting plants with CPMV. The entire aerial section of the plant was sprayed at each treatment. Short-term oxidative stress was simulated by treating plants for 2 days after infection with CPMV. Long-term oxidative stress was simulated by treating plants for 14 days after infection with CPMV. Optionally, ROS stress was combined with temperature stress by cultivating plants at a temperature of 30 °C instead of 25 °C for the entire duration of the experiment.

### Chlorophyll measurements

The leaf chlorophyll content was estimated non-destructively using a SPAD-502 chlorophyll meter (Minolta) after 2 weeks of exposure to simulated space conditions (i.e., microgravity and optional oxidative stress and change in temperature). The average function of the device was used to calculate the chlorophyll content from six distinct spots on the top side of leaves, avoiding major veins. Before each use, the chlorophyll meter was calibrated according to the manufacturer’s instructions.

### Extraction with a blender

Extracts of whole leaves were prepared with a blender, using three volumes (3 v/m) of extraction buffer (0.1 M potassium phosphate, pH 7.0) as previously described^48^. Before analysis, extracts were filtered through two layers of Miracloth (MilliporeSigma), and clarified by centrifugation at 16,000 × *g* for 30 min at 4 °C using an Avanti J-E centrifuge and a JLA 16.250 rotor (Beckman Coulter).

### Infiltration-centrifugation

The infiltration-centrifugation technique was used to extract apoplastic fluid from plant leaves^23^. Briefly, freshly harvested leaves were infiltrated with buffer (Table 1) by applying vacuum (70 cm Hg) for 2 min, followed by a rapid release of the vacuum^49^. Leaves were then carefully dried with paper towels and the infiltrated buffer was recovered by centrifugation at 1000 × *g* for 10 min at room temperature^23^. The eluate was either analyzed directly or further purified for scale-up experiments.

**Table 1:** Buffers used for infiltration-centrifugation

Buffer	Concentration [M]	Extraction pH
Glycine	0.1	9.0
Potassium phosphate	0.1	7.0
MES	0.1	4.0

### Ultrafiltration/diafiltration (UF/DF)

All ultrafiltration experiments were carried out using a Minimate bench-top tangential flow (TFF) filtration system (Cytiva) with 50-cm² 300-kDa polyethersulfone membranes (Cytiva) at a transmembrane pressure of 0.5 bar and a flow rate of 40 mL/min. Filter-sterilized extraction buffer (0.1 M potassium phosphate, pH 7.0) was used for all experiments. Ultrafiltration membranes were regenerated by rinsing with 4 mL/cm² extraction buffer and 4 mL/cm² 1.0 M sodium hydroxide, followed by incubation overnight as previously described^50^. Residual sodium hydroxide was removed by washing with 4 mL/cm² deionized water and 4 mL/cm² 20% (v/v) ethanol. The latter was also used for the storage of membranes.

### Bicinchoninic acid (BCA) assay

The concentration of total soluble protein in process samples was quantified with the Pierce BCA Protein Assay Kit (Thermo Fisher Scientific) using eight bovine serum albumin (BSA) standards in the range 25–2000 mg/L. Samples and standards were measured in triplicate.

### Enzyme-linked immunosorbent assay (ELISA)

The concentration of CPMV in process samples was quantified using a double antibody sandwich enzyme-linked immunosorbent assay (DAS-ELISA) kit (Agdia) with six CPMV standards at concentrations of 0.0–0.6 µg/mL. Deviating from the manufacturer’s instructions, plates were blocked with 200 µL 3% (w/v) BSA before adding standards or samples (100 µL) to the wells. All incubation steps were carried out at 25 °C on a rotary shaker at 100 rpm for 1 h. Between incubation steps, plates were washed three times with 200 µL PBS-T (PBS pH 7.4 containing 0.05% (v/v) Tween 20). Samples and standards were measured in triplicate.

### Sodium dodecylsulfate polyacrylamide gel electrophoresis (SDS-PAGE)

Process samples were separated on 4–12% Bis-Tris gels in MOPS buffer (Thermo Fisher Scientific) at 200 V for 45 min. Gels were subsequently stained with Coomassie Brilliant Blue or used for western blotting as previously described^44^. CPMV was detected using a rabbit anti-CPMV primary antibody (custom-made, Pacific Immunology) and an HRP-conjugated goat anti-rabbit secondary antibody (Thermo Fisher Scientific) as previously described^50^. The primary antibody was diluted 1:500 in 5% (m/v) milk powder in PBST, and the secondary antibody was diluted 1:5000 in the same buffer^51^. Gels were scanned with a FluorChem R system (ProteinSimple) followed by densitometric analysis with the on-board software.

### Transmission electron microscopy (TEM)

Process samples were analyzed by TEM using 400-mesh formvar/carbon-supported copper grids (Electron Microscopy Sciences) charged with the PELCOeasiGlow system (Ted Pella). Grids were negatively stained with 2.0% (w/v) uranyl acetate (Agar Scientific) and imaged at 20,000× or 80,000× magnification and 80 keV using a JEOL 1400Plus transmission electron microscope as previously described^50^.

### Statistics

Datasets were tested for normality using a Shapiro-Wilk test in GraphPad Prism v10 (GraphPad). Differences in plant health (chlorophyll content) or CPMV accumulation between simulated space conditions and static ground controls (1 × *g*) were evaluated by two-way analysis of variance (ANOVA) using GraphPad Prism v10 with a post hoc Tukey or Šidák test^52^ and a significance level of *α* = 0.05. If a significant factor interaction was found, only simple main effects were analyzed. In comparisons between two groups, N denotes the combined sample size across both groups, whereas n represents the sample size within one group.

## Results

### CPMV nanoparticles can be eluted from the apoplast without disrupting plant tissue

**Fig. 1:**
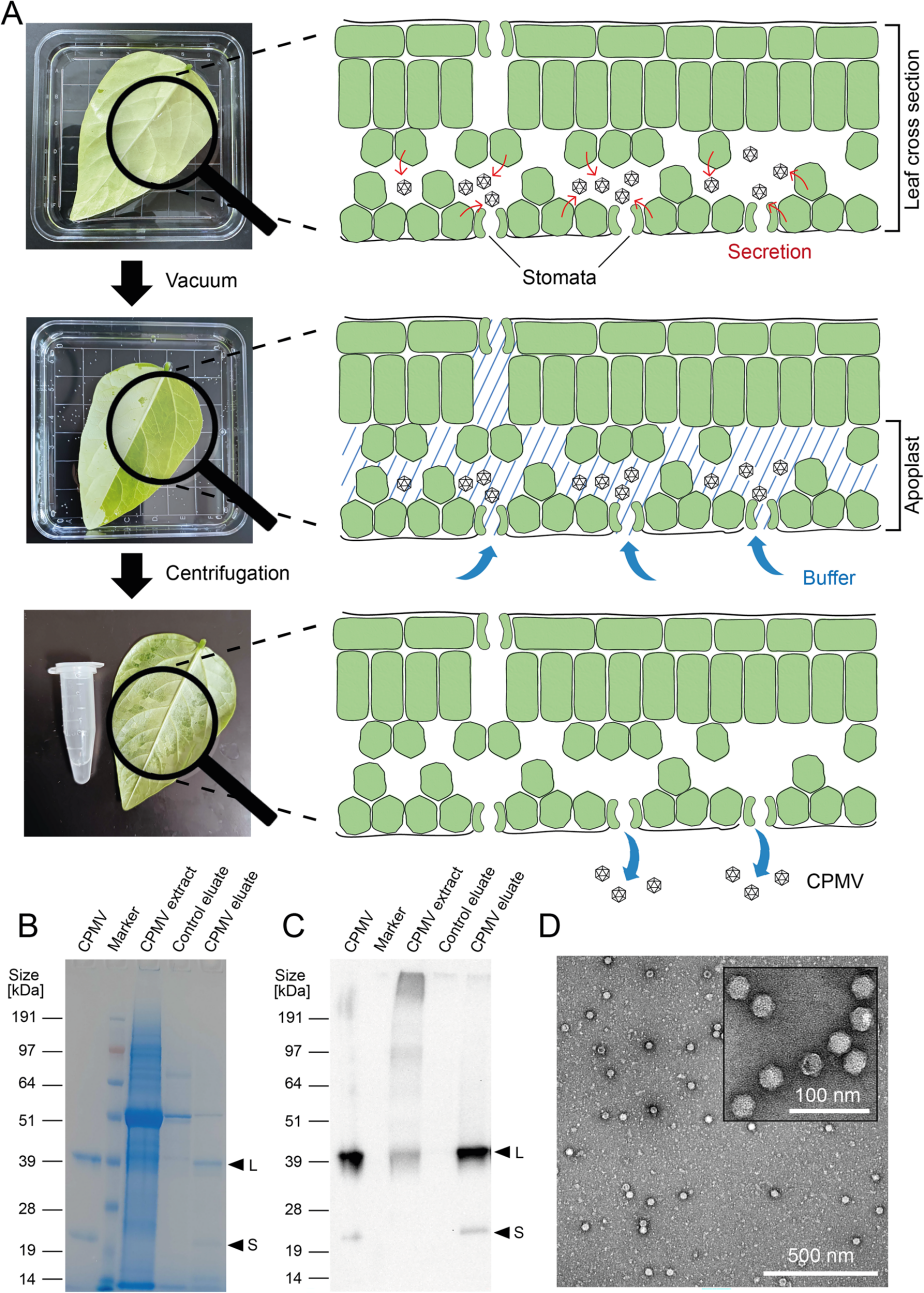
Strategy for non-disruptive CPMV extraction from leaves and analysis of apoplast eluates. **A** Elution of CPMV from the apoplast using the infiltration-centrifugation method. Leaves are submerged in buffer and a vacuum is applied to infiltrate the apoplastic space with buffer. The infiltrated buffer is recovered by centrifugation. Leaf cross sections are not drawn to scale. Note that legumes have stomata on both the adaxial and abaxial leaf surfaces, with the majority concentrated on the abaxial surface^105^. Analysis of apoplast eluates and blender extracts by SDS-PAGE followed by staining with Coomassie Brilliant Blue (**B**) or western blotting (**C**). Apoplast eluates were collected from CPMV-infected plants and non-infected control plants. Rabbit anti-CPMV antibodies and HRP-labeled goat anti-rabbit antibodies were used for western blotting. Black arrows denote the large (L) and small (S) CPMV coat proteins. **D** TEM images of apoplast eluates showing intact icosahedral particles.

To determine whether CPMV accumulates in the apoplast, allowing nondestructive recovery, we mechanically inoculated black-eyed pea plants with wild-type CPMV and extracted the apoplastic fluid from infected leaves by infiltration-centrifugation^23^. The leaves were submerged in buffer (0.1 M potassium phosphate, pH 7.0) and a vacuum was applied then rapidly released to drive infiltration into the apoplastic space (Fig. 1A). The infiltrated buffer was then recovered from the leaves by centrifugation (Fig. 1A). We describe these process samples as eluates to distinguish them from extracts prepared by disrupting plant tissue with a blender.

We compared the nondestructive eluates and blender extracts by SDS-PAGE followed by staining with Coomassie Brilliant Blue (Fig. 1B) and western blotting (Fig. 1C). The small (24 kDa) and large (42 kDa) CPMV coat proteins were the dominant proteins in apoplast eluates, as confirmed by western blotting with anti-CPMV antibodies. The purity of CPMV was ~200-fold higher in the apoplast eluates (~20%) than the blender extracts (~0.1%) based on densitometric analysis because major plant host cell proteins (HCPs) were absent (Fig. 1B). SDS-PAGE also revealed a greater number of HCPs in eluates from healthy control plants vs CPMV-infected plants (Fig. 1B), indicating that CPMV infection changes the abundance of plant HCPs in the apoplast. Importantly, transmission electron microscopy (TEM) confirmed that CPMV recovered from the apoplast is assembled into intact particles (Fig. 1D).

### Simulated space conditions affect plant morphology and plant physiology

**Fig. 2:**
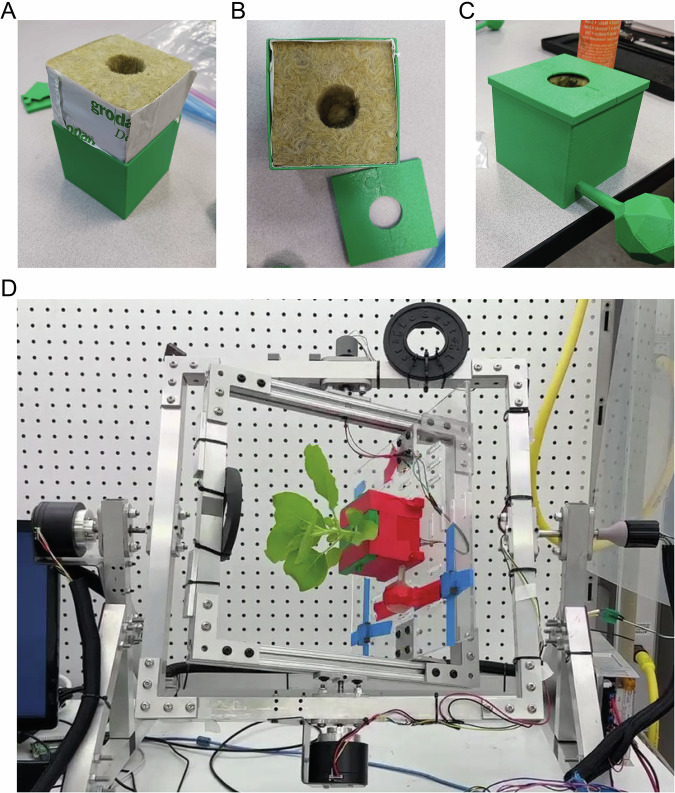
Setup for simulating microgravity on the ground using a customized random positioning machine. **A**, **B** Plants were grown in stone wool blocks to simulate growth conditions on spacecraft. Stone wool blocks were encased in 7.5 × 7.5 × 6.5 cm (*L* × *W* × *H*) plastic molds to simulate root zone hypoxia. **C** A water reservoir was attached to the plastic mold for irrigation. **D** The random positioning machine was housed in a walk-in growth chamber to allow the precise control of temperature, humidity and light intensity during experiments.

**Fig. 3:**
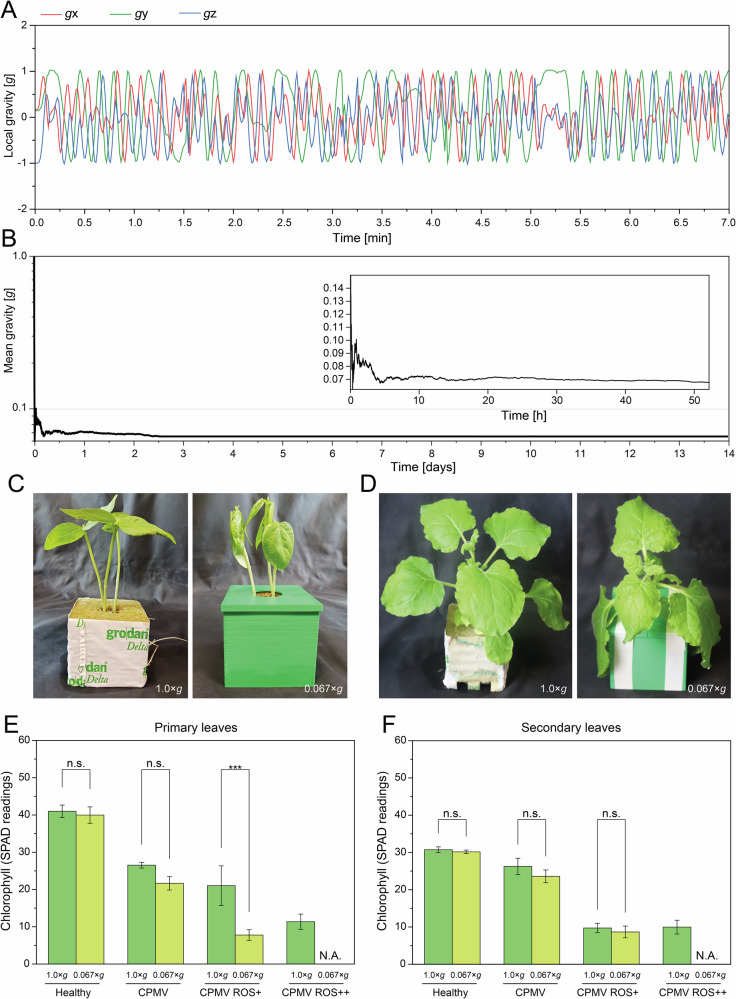
Effect of simulated space conditions on the plant host. Simulation of microgravity using a random positioning machine. **A** Local gravity vectors were measured with an accelerometer. **B** The mean gravity was calculated from the vector summation of the local gravity vectors^53^. Plant morphology under simulated space conditions. Representative images of black-eyed peas (**C**) and *N. benthamiana* (**D**) after 5 days under simulated space conditions. Plant physiology under simulated space conditions. The chlorophyll content was measured in primary (**E**) and secondary (**F**) leaves of uninfected and CPMV-infected black-eyed pea plants after 14 days under simulated space conditions. ROS treatment started 2 days before infecting primary leaves with CPMV and continued until 2 days post-infection (dpi) for the short-term treatment group and until 14 dpi for the long-term treatment. All plants were grown at 25 °C. Error bars represent the standard deviation from *n* = 3–4 individual plants with six technical replicates per measurement (*p < 0.05, **p < 0.01, ***p < 0.001, n.s. = not significant). ROS^+^ short-term ROS stress (2 days), ROS^++^ long-term ROS stress (14 days). N.A. not available.

Having established that intact CPMV particles can be eluted from the apoplast, we investigated the effect of a simulated space environment on this process. To simulate microgravity, we customized a RPM for plants (Fig. 2**;** Supplementary Movie 1). The mean effective gravity in the RPM was 0.067 × *g* (Fig. 3A, B), comparable with similar setups for earth-bound spaceflight experiments^53^. Because light acts as a substitute for gravity in growth regulation^54,55^, the light source was mounted outside of the RPM, resulting in continuously changing angles of light incidence. To mimic ROS stress caused by cosmic radiation, plants grown under terrestrial conditions (1 × *g*), as well as plants grown under microgravity (0.067 × *g*), were sprayed with 100 µM hydrogen peroxide three times per day^47^. ROS treatment started 2 days before the infiltration of primary leaves with CPMV and continued until 2 days post infection (dpi) for the short-term treatment group, and until 14 dpi for the long-term treatment group.

We first evaluated non-infected control plants to establish baseline growth and morphology. Compared with 1 × *g* controls, black-eyed pea plants grown under simulated microgravity exhibited a compact, spherical morphology (Fig. 3C), a phenotype that was reproducible in *N. benthamiana* (Fig. 3D). We next assessed whether simulated microgravity and ROS stress had an impact on plant health; we used leaf chlorophyll content as a proxy for photosynthetic function and plant health. Simulated microgravity alone had no significant effect on chlorophyll levels in healthy black-eyed pea plants (Fig. 3E, F; Supplementary Tables 1, 2). CPMV infection (a biotic stressor) significantly reduced the chlorophyll content in both primary (ANOVA, *p* < 0.0001, *N* = 7, *α* = 0.05) and secondary leaves (ANOVA, *p* = 0.004, *N* = 7, *α* = 0.05) compared with healthy controls (Fig. 3E, F; Supplementary Tables 1, 2). The addition of ROS, a known abiotic stressor, reduced chlorophyll levels more significantly, and statistical analysis indicates a significant interaction among CPMV infection, ROS stress, and simulated microgravity (ANOVA, *p* = 0.001, *N* = 21, *α* = 0.05; Fig. 3E; Supplementary Table 1).

### Apoplastic CPMV accumulation increases under ROS and temperature stress

Next, we determined whether ROS and temperature stressors would impact CPMV yields. We exposed plants to simulated short-term and long-term ROS stress, and also tested ambient (25 °C) and elevated (30 °C) temperatures—the latter allows us to mimic temperature fluctuations observed in extraterrestrial growth systems due to often occurring technical problems^56^. Again, short-term and long-term ROS stress was considered (as described above). We conducted these experiments under regular Earth gravity (1 × *g*) only. A DAS-ELISA was used to quantify CPMV in blender extracts and apoplast eluates^57^.

**Fig. 4:**
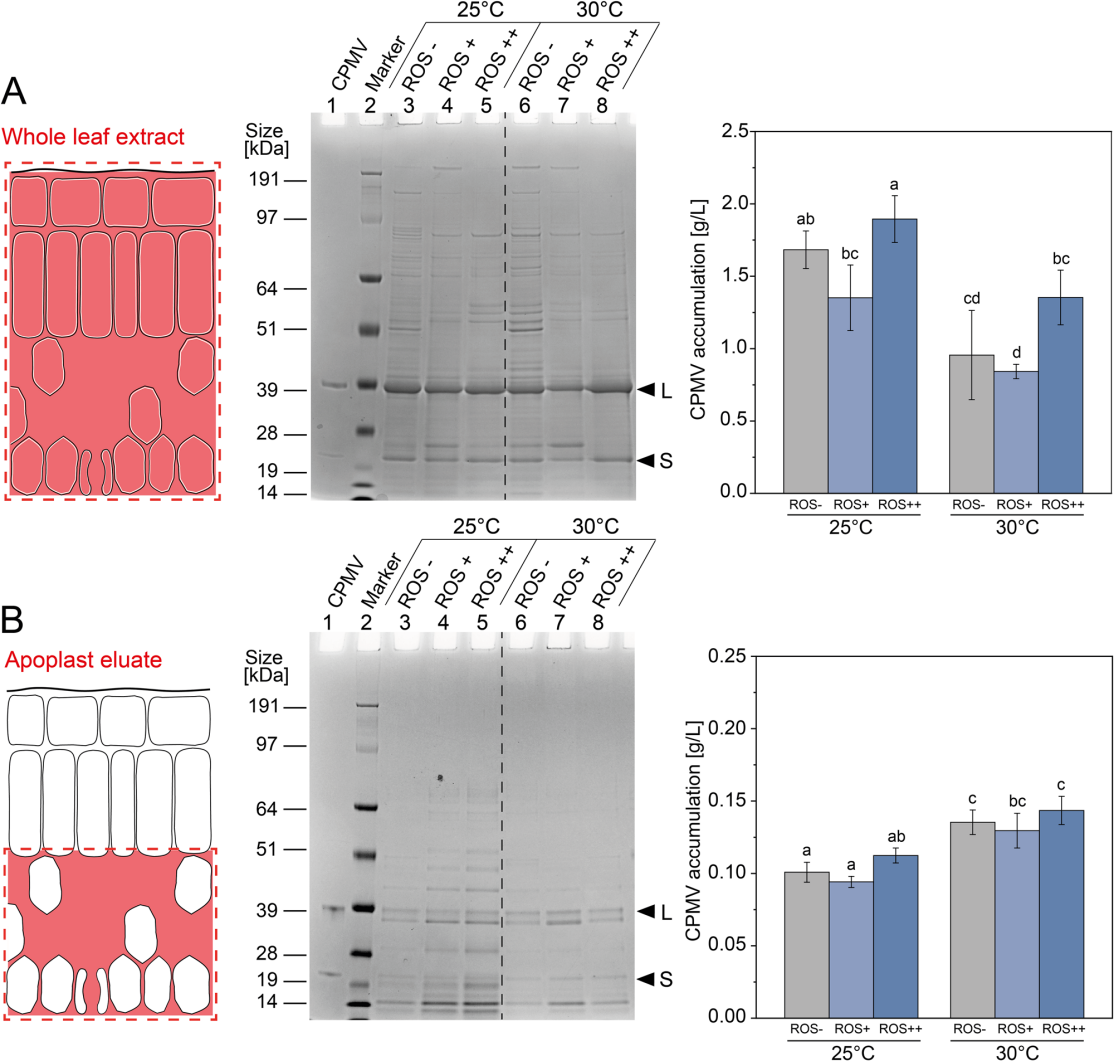
Effect of simulated space conditions on CPMV accumulation in black-eyed pea plants. **A** Effect of temperature and ROS stress on CPMV accumulation in blender extracts of primary leaves. ROS treatment started 2 days before infecting primary leaves with CPMV and continued until 2 days post-infection (dpi) for the short-term treatment group and until 14 dpi for the long-term treatment group. Plants were grown under terrestrial conditions (1 × *g*). Blender extracts were analyzed by gel electrophoresis and staining with Coomassie Brilliant Blue. CPMV was quantified using a commercial DAS-ELISA kit. **B** Effect of temperature and ROS stress on CPMV accumulation in apoplast eluates of primary leaves. Experiments were conducted and analyzed as described above. Error bars represent the standard deviation from *n* = 3–4 batches of 12 plants per batch. Lowercase letters indicate significance groups (Table S3, Table S4). Conditions that share the same letter were not significantly different (*p* > 0.05). ROS^–^ = no stress, ROS^+^ = short-term ROS stress, ROS^++^ = long-term ROS stress.

Blender extracts of primary leaves contained ~2.0 g/L CPMV at 25 °C. Although symptoms appeared earlier at 30 °C (Supplementary Fig. 1), constant exposure to this elevated temperature significantly reduced CPMV yields in blender extracts by 35.4 ± 14.4% across all treatments (Fig. 4A**;** Supplementary Table 3). In contrast, apoplast eluates showed the opposite trend: CPMV levels significantly increased by 33.8 ± 3.6% at 30 °C in primary leaves (Fig. 4B; Supplementary Table 4). This increase was accompanied by more severe infection symptoms (Fig. S2) and an ~80% reduction in host cell proteins compared to 25 °C (Fig. 4B). Overall, CPMV yields decrease with elevated temperature in blender extracts but increased in apoplast eluates. Thus, we concluded that ~25 °C is optimal for maximizing yields in blender extracts, while 30 °C is optimal maximizing CPMV yields in apoplast eluates. We note that overall blender extracts result in higher yields compared to CPMV eluted from the apoplast (see also below).

The effect of ROS on CPMV yields was subtle: while short-term ROS exposure led to lower CPMV levels, long-term ROS exposure increased CPMV yield. Short-term exposure to ROS likely primes plant defense systems^58^, reducing CPMV infection, while long-term ROS stress weakens the overall health status of the plant, allowing for more efficient CPMV infection. Trends were similar comparing the blender extracts and apoplastic eluate (Fig. 4; Supplementary Tables 3+4).

### Elution of CPMV from the apoplast is scalable

**Fig. 5:**
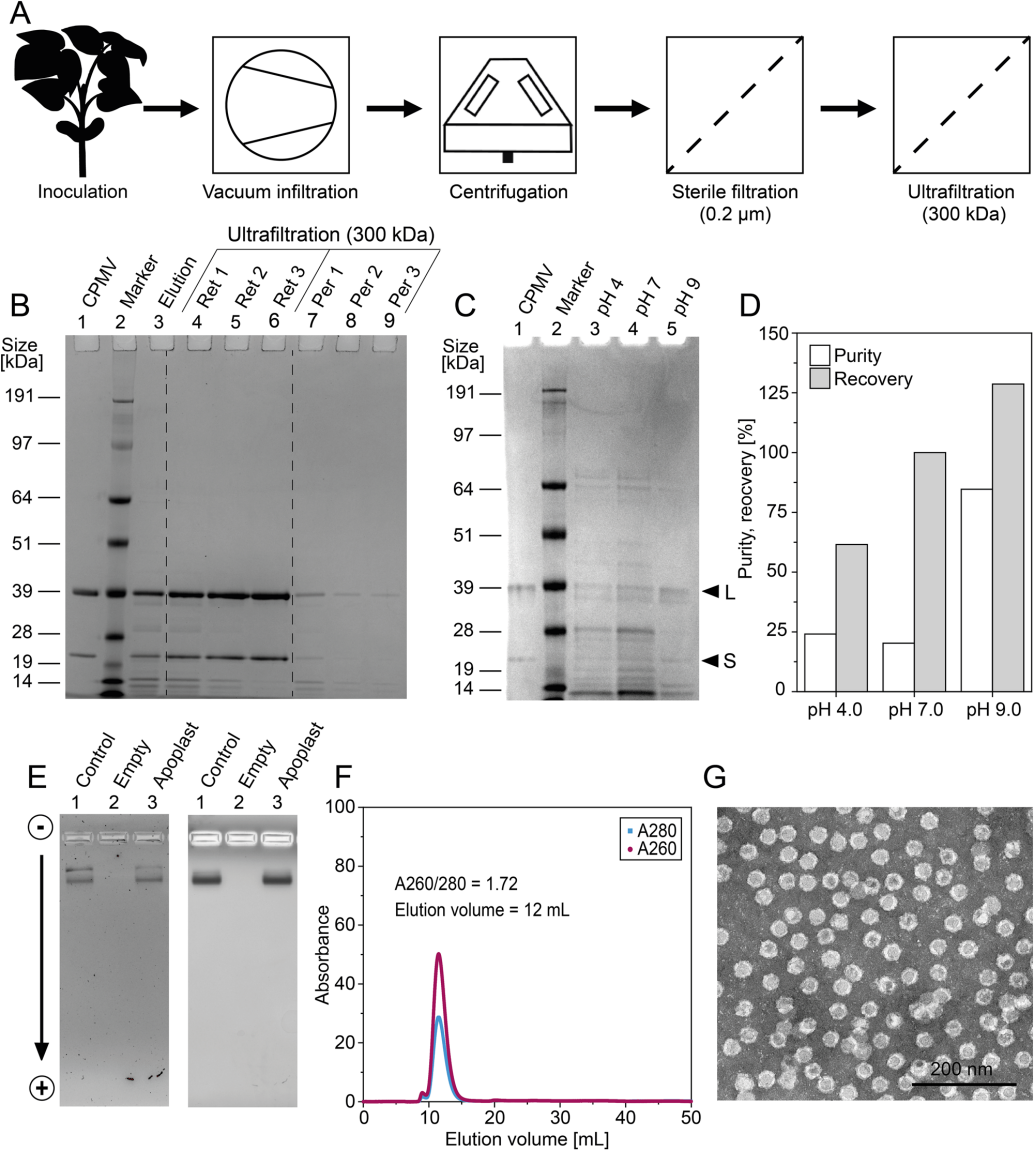
Process scale-up, optimization and characterization. **A** Process flow diagram for the simplified purification of CPMV from plants by infiltration-centrifugation and UF/DF. **B** Analysis of process samples from the same process. Samples were separated by gel electrophoresis and stained with Coomassie Brilliant Blue. UF/DF retentates are concentrated two-fold compared to the other process samples. Analysis of apoplast eluates from CPMV-infected black-eyed pea plants using alkaline, neutral and acidic buffers by denaturing gel electrophoresis (**C**) and staining with Coomassie Brilliant Blue. Gels were imaged with a FluorChem R system (ProteinSimple), followed by densitometric analysis (**D**) with on-board software. **E** Native gel electrophoresis of CPMV preparations followed by RNA staining (left) and protein staining (right), Lane 1 = Control CPMV purified from blender extracts, lane 2 = empty, lane 3 = CPMV purified from apoplast eluates. **F** SEC elution profile and **G** TEM image of CPMV purified from apoplast eluates at pH 7.0.

To assess the scalability of CPMV production in the apoplast, we tested our simplified production process with more than 50 plants at a time grown at 1 × *g*. To remove remaining impurities in apoplast eluates, we combined the infiltration-centrifugation method with ultrafiltration/diafiltration (UF/DF) to ensure high purity (typically ≥99%^59^), which is a prerequisite for the clinical application of plant virus-based therapeutics. The resulting process had five steps, which can be completed in less than 2 h (Fig. 5A).

We found that UF/DF with a 300-kDa molecular weight cut-off (MWCO) membrane retained the majority of CPMV, whereas impurities were washed out in the permeate (Fig. 5B). The purity of CPMV preparations from infiltration-centrifugation and UF/DF was >99%, thus matching the purity threshold for clinical applications. The process yield was 0.49 mg from 100 g of leaves, which is ~40× lower compared to purification of CPMV by blender extraction and UF/DF^50^. While yields are lower compared to blender extracts, the method would still be of value for space travel where low doses for a few patients would need to be produced. Furthermore, buffer recovery from leaves was only ~50% (Table 2), indicating that this step can be further optimized to maximize process yields. Additional considerations are laid out in the discussion section.

**Table 2:** Infiltration-centrifugation process metrics during scale-up

Leaf mass harvested [g]	Leaf mass after infiltration [g]	Leaf mass after centrifugation [g]	Volume apoplast eluate [mL]	Recovery infiltration buffer [%]
42.6 (per tray, 200 in², 0.129 m²)	75.1	52.8	17.5	54

One tray of plants corresponds to *n* = 18 pots with three plants each.

Taking advantage of the broad pH stability of CPMV (pH 4.0–9.0^60^), we evaluated acidic (pH 4.0), neutral (pH 7.0) and alkaline (pH 9.0) buffers for the elution of CPMV from the apoplast (Fig. 5C, D). We found that alkaline extraction buffers (pH 9.0) achieved a higher purity (85%), compared to neutral (20%) or acidic elution buffers (24%). Acidic extraction conditions were accompanied by a reduced recovery of CPMV (Fig. 5C, D), consistent with our previous work^50^.

### Characterization of CPMV after elution from the apoplast and purification by UF/DF

CPMV preparations from the infiltration-centrifugation and UF/DF purification processes were characterized, to confirm the purity and integrity of the particles, by native agarose gel electrophoresis, size exclusion chromatography (SEC) and TEM. Native gel electrophoresis confirmed the co-localization of the CPMV RNA and coat protein, confirming the CPMV particles were intact (Fig. 5E). Typical CPMV preparations obtained from blender extracts contain slow and fast electrophoretic forms of CPMV reflecting a mixture of particles with cleaved and uncleaved S protein^61^. CPMV purified from the apoplast predominantly consisted of the fast electrophoretic form, consistent with the cleavage of the S protein under acidic conditions of the apoplast^50^. SEC revealed the typical profiles of intact CPMV preparations eluting at ~12 mL from a Superose 6 Increase column with an A260/280 ratio of ~1.7^50^. There were no signs of aggregation, degradation or impurities, indicating that the latter were efficiently removed during UF/DF with a 300-kDa MWCO membrane (Fig. 5F). TEM imaging confirmed that CPMV from the infiltration-centrifugation and UF/DF purification processes was uniform and intact (Fig. 5G). Overall, the data confirmed that our simplified purification process can produce intact and pure CPMV.

## Discussion

The purification of recombinant proteins from plants usually involves the disruption of plant tissue^62^, resulting in the release of large quantities of soluble and insoluble impurities^63,64^. In contrast, animal cells and microbes often secrete recombinant proteins into the cultivation medium, which facilitates purification^62^. In plants, the presence of a cell wall external to the plasma membrane causes secreted proteins to accumulate in a compartment known as the apoplast, necessitating additional processing steps for efficient recovery. Here, we implemented an infiltration-centrifugation strategy to recover CPMV from the apoplast of whole plants, which would allow the production and utilization of CPMV-based therapeutics in low-resource environments such as long-term space missions. Because the plant tissue was not destroyed when extracting CPMV, repeated harvest cycles become feasible, thus increasing resource efficiency and sustainability^24^.

CPMV has not previously been observed in the apoplast, but potato virus X (PVX) has been detected in this compartment^65,66^ and brome mosaic virus among others has been detected in guttation samples^67–69^, indicating a precedent for the elution of plant viruses from extracellular compartments and subsequent purification by UF/DF. Accordingly, we found that intact CPMV particles were present in the apoplast and could be eluted by centrifugation without disrupting the plant tissue, increasing the purity of CPMV preparations ~200-fold compared to blender extracts. This confirms that the plant tissue was not damaged during the infiltration-centrifugation process, because damage would cause the release of intracellular HCPs, including the hyperabundant ribulose-1,5-bisphosphate carboxylase/oxygenase (RuBisCO), which were indeed detected in the blender extracts. Alkaline extraction buffers were the most effective, achieving a CPMV purity of 85% compared to 20–24% for neutral and acidic buffers. This result may appear surprising at first because acidic buffers remove plant HCPs efficiently from extracts of whole plants^50^. However, unlike the cytosol of plant cells, which has a neutral or slightly alkaline pH^70^, the apoplast is an acidic environment with a pH as low as 4.5^71,72^. HCPs in this compartment should be accustomed to acidic conditions and would thus be removed inefficiently. The recovery of CPMV was also lower with acidic buffers, as we previously reported^50^, probably reflecting the low solubility of CPMV close to its isoelectric point of 5.5^73^. In contrast, the higher recovery of CPMV with alkaline buffers may reflect the higher net negative surface charge, increasing electrostatic repulsion and thus reducing protein aggregation and minimizing nonspecific interactions.

CPMV infection also reduced the abundance of HCPs in the apoplast compared to uninfected plants. This observation is consistent with literature investigating the effect of PVX infections on the apoplast proteome of *N. benthamiana*^66^. The suppression of HCP expression and/or secretion further simplifies the purification of virus particles from the apoplast and is thus synergistic with our aim to simplify plant-based purification processes. To the best of our knowledge, this is the first time that apoplast recovery has been used as a strategy to purify CPMV (or other plant viruses) from plants. Remaining impurities in apoplast eluates were readily removed by UF/DF, exploiting the size difference between CPMV and impurities. Specifically, CPMV has a molecular mass of 5.6 × 10^3 ^kDa^74^, thus exceeding the molecular mass of even the largest HCPs (<50 kDa^75^), as well as metabolites such as phenolics (~5 kDa^76^) and simpler pigments (~1 kDa^77^) by several orders of magnitude.

Although the current process produces sufficient material for a low number of doses and limited number of patients on a spacecraft, further optimization would make this process highly attractive for low-resource settings on Earth. To improve overall process yields, we will next optimize the recovery of infiltrated buffer from leaves, which was only ~50% with our current setup. This could be achieved for example by varying the duration and gravitational force of the centrifugation step, using statistical design of experiments for multi-factor optimization. Importantly, our elution-based process enables repeated elution cycles with the same leaf material. This strategy has been shown to increase product yields at least two-fold compared to a single elution cycle^25^. An alternative strategy to improve process yields is to combine elution-based downstream processing with genetic strategies to enhance the secretion of proteins to the apoplast; for example secretion signal sequences could be appended to the CPMV coat proteins^78–80^. One could also consider novel strategies such as modification of key components of the secretory pathway to enhance protein trafficking in plants^81,82^. For instance, a combination of ER engineering with chaperone overexpression increased the yields of assembled secretory immunoglobulins by an order of magnitude in *N. benthamiana*^81^.

A key barrier that must be overcome before plants can be used as a source of pharmaceuticals during space missions is their adaptation to the unique forms of stress found in extraterrestrial environments. Major stressors that affect plants in space include low gravity and cosmic radiation^83^, both of which generate ROS^84^. Although space flight-induced stress has mainly been perceived as a threat to plants, our data show that it can also provide an opportunity to increase productivity in the context of plant molecular farming. For example, we found that microgravity induced a compact, spherical morphology, which could in theory be exploited to grow more plants in a limited growth area, ultimately saving mission resources. Specifically, the ideal plant form for growth in space settlements is compact with a canopy structure that maximizes photosynthesis^85^. Controlling plant morphology through cultivation conditions could complement genetic engineering approaches to achieve the ideal plant form^86^. In contrast, we found that microgravity had no significant effect on chlorophyll levels, so additional stressors may be required to elicit significant physiological changes^87^.

We found that CPMV accumulation in plants decreased after short-term exposure to ROS stress but increased during long-term exposure. The dual role of ROS in virus resistance and susceptibility is consistent with the literature^88^. Reduced CPMV titers after short-term exposure to ROS stress may reflect the activation of antioxidant and pathogenesis-related genes in plants at low concentrations of hydrogen peroxide^88–90^. For example, ROS stress induces peroxidase-mediated cross-linking of cell wall proteins, thus reinforcing the latter against infections. Hydrogen peroxide is also thought to induce defense genes during infection^91^. Low concentrations of exogenously applied hydrogen peroxide have been shown to prime plants against various abiotic^92,93^ and biotic stressors^94–96^.

A potential explanation for the increased accumulation of CPMV after long-term exposure to ROS is that ROS may promote viral replication by mediating the oxidation of viral factors^88^. Additionally, the oxidative inactivation of ribonucleotide reductase, which catalyzes the rate-limiting step of DNA synthesis^97^, may favor the replication of RNA viruses^88^ such as CPMV^98^. On spacecraft, plants are expected to experience long-term oxidative stress^46^, and our data suggest that this may increase CPMV yields. From a process resilience perspective, the production of CPMV by plant molecular farming is therefore well-suited for space missions.

In addition to ROS, we also observed a significant effect of temperature on CPMV yields. The temperature-dependence of virus–host interactions is consistent with the literature^99,100^, and has previously been linked to the activity of heat shock proteins^101^. Notably, the effect of elevated temperatures on virus accumulation appears to be time-dependent^99,100^. Specifically, short-term exposure to elevated temperatures often increases virus titers, whereas long-term exposure reduces virus accumulation in plants^99^. Other factors that may influence virus accumulation include the plant growth stage, light intensity, air movement and nutrition, highlighting the need to carefully control for all variables in experiments^100^. Temperature not only affected virus yields but also purity because HCPs in apoplast eluates were depleted by ~80% at 30 °C compared to 25 °C. These results show that apoplastic virus accumulation does not necessarily mimic virus accumulation in blender extracts, and that both methods should be compared carefully^67^.

Unlike terrestrial stressors, which typically reduce recombinant protein expression in plants because they can induce stress-related proteins that compete with the recombinant protein machinery^102–104^, space-flight induced stress improved the accumulation of CPMV in plants. The improved process resilience is highly desirable for applications in space and on the ground. For example, resilience in pharmaceutical production is one of the main objectives of the EU Horizon 2020 and Horizon Europe 2025–2027 programs, which are also aligned with the 2022 National Biotechnology & Biomanufacturing Initiative (NBBI) in the US.

Our findings demonstrate that integrating the recovery of proteins from the apoplast with a cultivation strategy that deliberately uses controlled stress (or responds dynamically to it) enhances both the efficiency and resilience of plant molecular farming. By enabling the gentle extraction of intact CPMV particles with enhanced purity, the infiltration-centrifugation approach simplifies downstream processing and enables repeated harvest cycles without the destruction of plant biomass. At the same time, controlled exposure to environmental stressors such as ROS and elevated temperature can modulate viral accumulation and purity in a predictable manner. The combination of process-level and host-level optimization facilitates sustainable CPMV production under the constrained conditions of long-duration space missions while also offering practical advantages for terrestrial biomanufacturing.

## Supplementary information

Supplementary information

Supplementary movie

## Data Availability

The data that support the findings of this study are available in datadryad at https://datadryad.org.
