# Supplementary material for: Streamlined molecular farming of plant virus therapeutics for space flight and other low-resource environments
Source: NPJ Sci Plants. 2026 Jun 5;2(1):16. doi: 10.1038/s44383-026-00030-y (PMC13241316; doi:10.1038/s44383-026-00030-y)
Supplement: Supplementary file 1 — Supplementary information [file 44383_2026_30_MOESM1_ESM.docx]

**Supplementary information for**

**Streamlined molecular farming of plant virus therapeutics for space flight and other low-resource environments**

Patrick Opdensteinen^[a,d,h]^, Kyle Lewin^[a,d,h]^, Anshal Jain^[i]^, Andrew Copeland^[j]^, Jonathan Copeland^[j]^, Maziar Ghazinejad^[j]^, Nicole F. Steinmetz^[a, b, c, d, e, f, g, h]^*

[a] Aiiso Yufeng Li Family Department of Chemical and Nano Engineering, University of California, San Diego, La Jolla, CA 92093, USA

[b] Department of Bioengineering, University of California, San Diego, La Jolla, CA 92093, USA

[c] Department of Radiology, University of California, San Diego, La Jolla, CA 92093, USA

[d] Center for Nano-ImmunoEngineering, University of California, San Diego, La Jolla, CA 92093, USA

[e] Institute for Materials Discovery and Design, University of California, San Diego, La Jolla, CA 92093, USA

[f] Moores Cancer Center, University of California, San Diego, La Jolla, CA 92093, USA

[g] Center for Engineering in Cancer, Institute of Engineering in Medicine, University of California, San Diego, La Jolla, CA 92093, USA

[h] Shu and K.C. Chien and Peter Farrell Collaboratory, University of California, San Diego, La Jolla, CA 92093, USA

[i] Department of Electrical and Computer Engineering, University of California, San Diego, La Jolla, CA 92093, USA

[j] Department of Mechanical and Aerospace Engineering, University of California, San Diego, La Jolla, CA 92093, USA

* Corresponding author: nsteinmetz@ucsd.edu

ORCID: https://orcid.org/0000-0002-0130-0481

**Supplementary Table 1: Comparison of chlorophyll levels in primary leaves (Figure 3E).**

Two-way ANOVA

|  | **Sum of squares** | **Degrees of freedom** | **Mean sum of squares** | **F-statistic** | **P-value** |
| --- | --- | --- | --- | --- | --- |
| Interaction | 167.90 | 2 | 83.96 | 11.44 | 0.0010 |
| CPMV & ROS | 2431.00 | 2 | 1215.00 | 165.50 | <0.0001 |
| Gravity | 187.20 | 1 | 187.20 | 25.50 | 0.0001 |
| Residual | 110.1 | 15 | 7.34 |  |  |

The interaction between ‘CPMV & ROS’ and ‘gravity’ is statistically significant.

Differences between gravity settings within each group (adjusted using Šidák correction)

|  | **Mean diff.** | **95.00% CI of diff.** | **Adjusted P-value** | **P-value summary** |
| --- | --- | --- | --- | --- |
| 1×g:Healthy vs. 0×g:Healthy | -0.02 | -5.57 to 5.54 | 0.9999 | ns |
| 1×g:CPMV vs. 0×g:CPMV | 4.87 | -1.07 to 10.81 | 0.1260 | ns |
| 1×g:CPMV ROS+ vs. 0×g:CPMV ROS+ | 13.25 | 8.11 to 18.39 | <0.0001 | **** |
| 1×g:Healthy vs. 1×g:CPMV | 13.42 | 8.28 to 18.56 | <0.0001 | **** |
| 1×g:Healthy vs. 1×g:CPMV ROS+ | 18.93 | 14.17 to 23.68 | <0.0001 | **** |
| 0×g:Healthy vs. 0×g:CPMV | 18.30 | 12.81 to 23.79 | <0.0001 | **** |
| 0×g:Healthy vs. 0×g:CPMV ROS+ | 32.19 | 27.05 to 37.33 | <0.0001 | **** |

The ‘CPMV ROS long’ group was omitted from the ANOVA because complete observations were not available.

**Supplementary Table 2: Comparison of chlorophyll levels in secondary leaves (Figure 3F).**

Two-way ANOVA

|  | **Sum of squares** | **Degrees of freedom** | **Mean sum of squares** | **F-statistic** | **P-value** |
| --- | --- | --- | --- | --- | --- |
| Interaction | 5.84 | 2 | 2.92 | 1.48 | 0.2594 |
| CPMV & ROS | 1638.00 | 2 | 818.80 | 414.90 | <0.0001 |
| Gravity | 8.29 | 1 | 8.29 | 4.20 | 0.0583 |
| Residual | 29.60 | 15 | 1.97 |  |  |

Differences between gravity settings within each group (adjusted using Šidák correction)

|  | **Mean diff.** | **95.00% CI of diff.** | **Adjusted P-value** | **P-value summary** |
| --- | --- | --- | --- | --- |
| 1×g:Healthy vs. 0×g:Healthy | 0.08 | -2.80 to 2.96 | 0.9998 | ns |
| 1×g:CPMV vs. 0×g:CPMV | 2.67 | -0.21 to 5.55 | 0.0737 | ns |
| 1×g:CPMV ROS+ vs. 0×g:CPMV ROS+ | 1.06 | -1.82 to 3.94 | 0.7119 | ns |
| 1×g:Healthy vs. 1×g:CPMV | 3.983 | 1.36 to 6.61 | 0.0040 | ** |
| 1×g:Healthy vs. 1×g:CPMV ROS+ | 20.52 | 17.89 to 23.14 | <0.0001 | **** |
| 0×g:Healthy vs. 0×g:CPMV | 6.567 | 3.96 to 9.17 | <0.0001 | **** |
| 0×g:Healthy vs. 0×g:CPMV ROS+ | 21.49 | 18.89 to 24.10 | <0.0001 | **** |

The ‘CPMV ROS long’ group was omitted from the ANOVA because complete observations were not available.

**Supplementary Table 3: Comparison of CPMV accumulation levels in blender extracts (Figure 4A).**

Two-way ANOVA

|  | **Sum of squares** | **Degrees of freedom** | **Mean sum of squares** | **F-statistic** | **P-value** |
| --- | --- | --- | --- | --- | --- |
| Interaction | 0.06 | 2 | 0.03 | 0.73 | 0.4945 |
| ROS | 1.12 | 2 | 0.56 | 14.77 | 0.0002 |
| Temperature | 2.11 | 1 | 2.11 | 55.53 | <0.0001 |
| Residual | 0.68 | 18 | 0.04 |  |  |

Multiple comparison (adjusted using Tukey correction)

|  | **Mean diff.** | **95.00% CI of diff.** | **Adjusted P-value** | **P-value summary** |
| --- | --- | --- | --- | --- |
| Control:25C vs. Control:30C | 0.73 | 0.29 to 1.17 | 0.0006 | *** |
| Control:25C vs. ROS short:25C | 0.33 | -0.11 to 0.77 | 0.2043 | ns |
| Control:25C vs. ROS short:30C | 0.84 | 0.40 to 1.28 | 0.0001 | *** |
| Control:25C vs. ROS long:25C | -0.21 | -0.65 to 0.23 | 0.6478 | ns |
| Control:25C vs. ROS long:30C | 0.33 | -0.11 to 0.77 | 0.2091 | ns |
| Control:30C vs. ROS short:25C | -0.40 | -0.83 to 0.04 | 0.0904 | ns |
| Control:30C vs. ROS short:30C | 0.11 | -0.32 to 0.55 | 0.9591 | ns |
| Control:30C vs. ROS long:25C | -0.94 | -1.38 to -0.50 | <0.0001 | **** |
| Control:30C vs. ROS long:30C | -0.40 | -0.84 to 0.04 | 0.0880 | ns |
| ROS short:25C vs. ROS short:30C | 0.51 | 0.07 to 0.95 | 0.0175 | * |
| ROS short:25C vs. ROS long:25C | -0.54 | -0.98 to -0.11 | 0.0104 | * |
| ROS short:25C vs. ROS long:30C | -1.98×10^-3^ | -0.44 to 0.44 | 0.9999 | ns |
| ROS short:30C vs. ROS long:25C | -1.05 | -1.49 to -0.61 | <0.0001 | **** |
| ROS short:30C vs. ROS long:30C | -0.51 | -0.95 to -0.07 | 0.0170 | * |
| ROS long:25C vs. ROS long:30C | 0.54 | 0.10 to 0.98 | 0.0107 | * |

**Supplementary Table 4: Comparison of CPMV accumulation levels in apoplast eluates (Figure 4B).**

Two-way ANOVA

|  | **Sum of squares** | **Degrees of freedom** | **Mean sum of squares** | **F-statistic** | **P-value** |
| --- | --- | --- | --- | --- | --- |
| Interaction | 1.49×10^-5^ | 2 | 7.46×10^-6^ | 0.11 | 0.8953 |
| ROS | 7.92×10^-4^ | 2 | 3.96×10^-4^ | 5.93 | 0.0162 |
| Temperature | 5.11×10^-3^ | 1 | 5.11×10^-3^ | 76.45 | <0.0001 |
| Residual | 8.02×10^-4^ | 12 | 6.68×10^-5^ |  |  |

Multiple comparison (adjusted using Tukey correction)

|  | **Mean diff.** | **95.00% CI of diff.** | **Adjusted P-value** | **P-value summary** |
| --- | --- | --- | --- | --- |
| Control:25C vs. Control:30C | -0.03 | -0.06 to -0.01 | 0.0025 | ** |
| Control:25C vs. ROS short:25C | 6.69×10^-3^ | -0.02 to 0.03 | 0.9086 | ns |
| Control:25C vs. ROS short:30C | -0.03 | -0.05 to -0.01 | 0.0102 | * |
| Control:25C vs. ROS long:25C | -0.01 | -0.03 to 0.01 | 0.5400 | ns |
| Control:25C vs. ROS long:30C | -0.04 | -0.07 to -0.02 | 0.0004 | *** |
| Control:30C vs. ROS short:25C | 0.04 | 0.02 to 0.06 | 0.0005 | *** |
| Control:30C vs. ROS short:30C | 5.81×10^-3^ | -0.02 to 0.03 | 0.9467 | ns |
| Control:30C vs. ROS long:25C | 0.02 | 5.60×10^-4^ to 0.05 | 0.0434 | * |
| Control:30C vs. ROS long:30C | -8.19×10^-3^ | -0.03 to 0.01 | 0.8161 | ns |
| ROS short:25C vs. ROS short:30C | -0.04 | -0.06 to -0.01 | 0.0020 | ** |
| ROS short:25C vs. ROS long:25C | -0.02 | -0.04 to 4.19×10^-3^ | 0.1394 | ns |
| ROS short:25C vs. ROS long:30C | -0.05 | -0.07 to -0.03 | <0.0001 | **** |
| ROS short:30C vs. ROS long:25C | 0.02 | -0.01 to 0.04 | 0.1780 | ns |
| ROS short:30C vs. ROS long:30C | -0.01 | -0.04 to 0.01 | 0.3494 | ns |
| ROS long:25C vs. ROS long:30C | -0.03 | -0.05 to -0.01 | 0.0055 | ** |


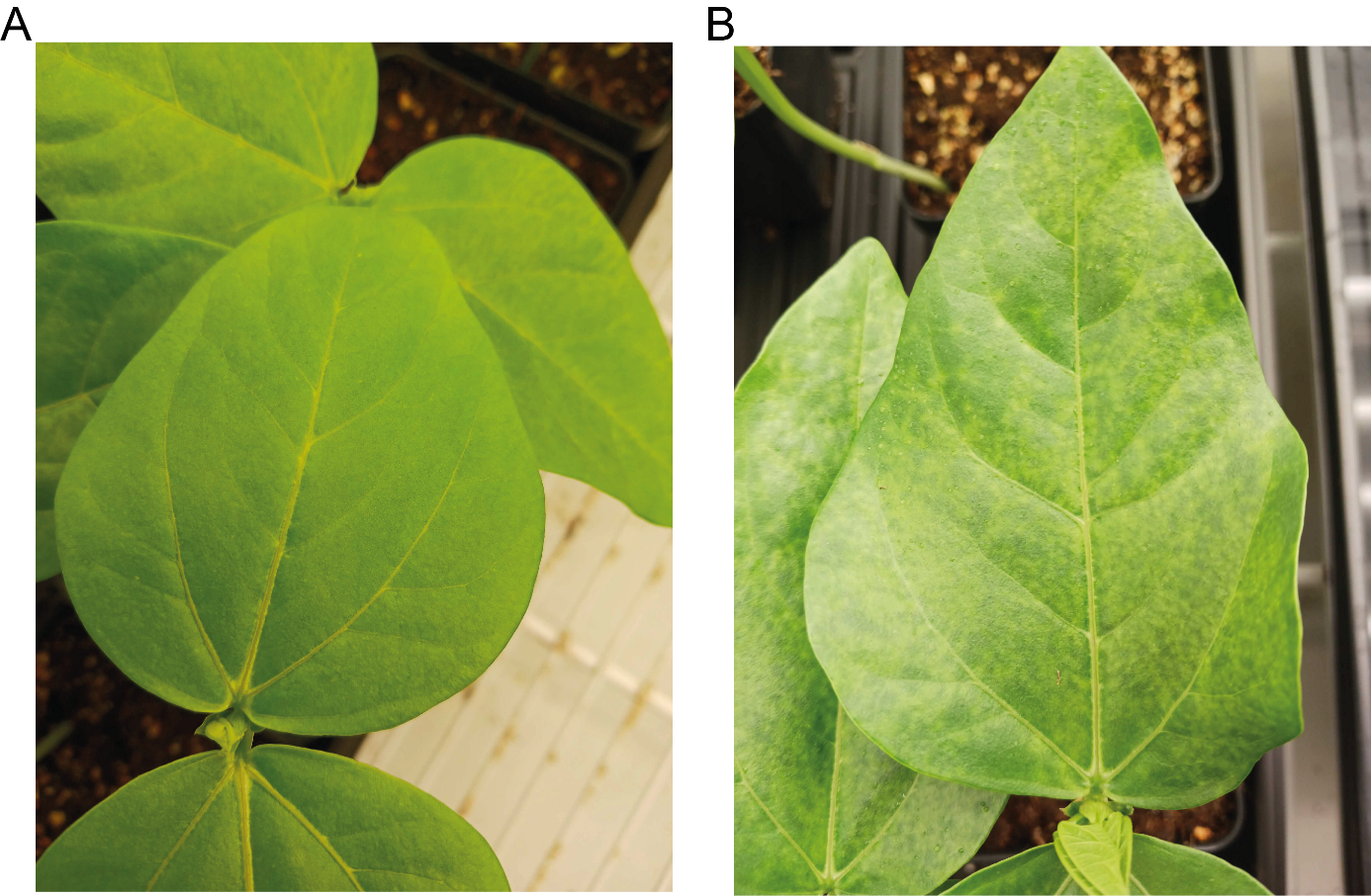


**Supplementary Figure 1: Effect of the incubation temperature on the onset of CPMV symptoms.** Representative images of primary leaves of black-eyed pea plants 3 days after mechanical inoculation with CPMV. Plants were incubated under regular Earth gravity at 25°C (**A**) or 30°C (**B**). Plants incubated at 30°C developed symptoms of CPMV infection before plants incubated at 25°C (after 3 days compared to 5–6 days for plants at 25°C).

**
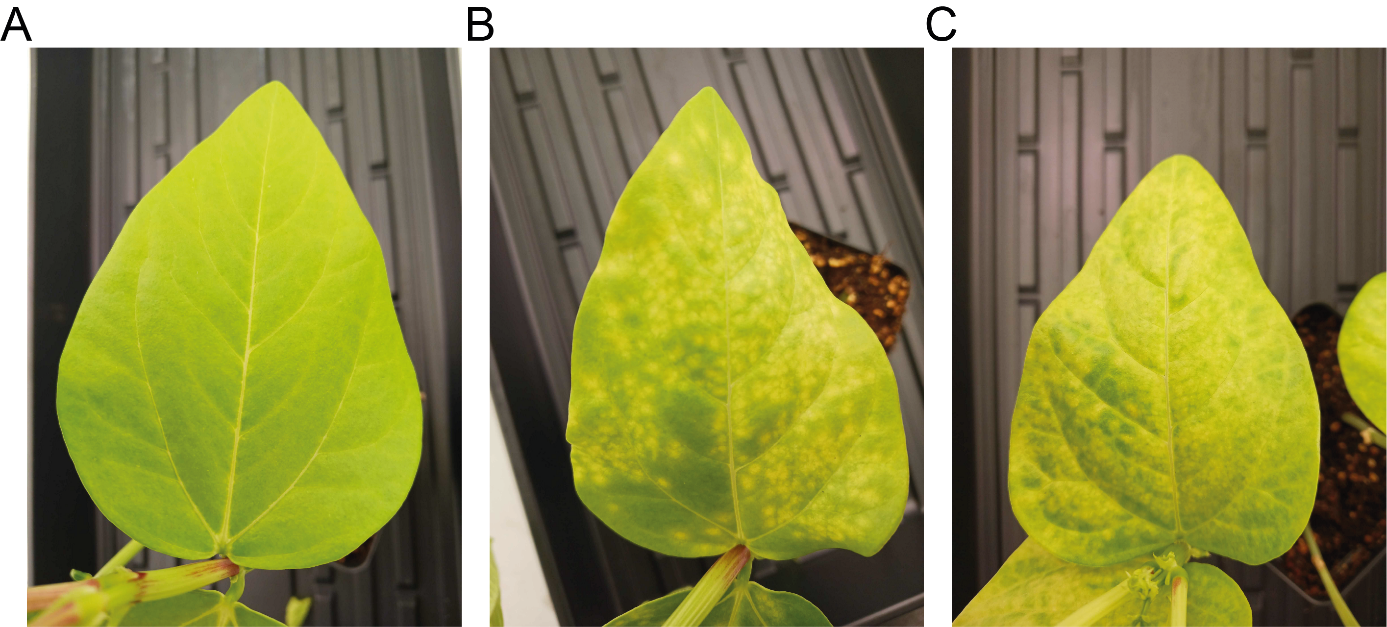
**

**Supplementary Figure 2: Effect of the incubation temperature on the severity of CPMV infection symptoms**. (**A-C**) Representative images of primary leaves of black-eyed pea plants 14 days after mechanical inoculation with CPMV. Plants were incubated under regular Earth gravity at 20°C (**A**), 25°C (**B**) or 30°C (**C**). Plants incubated at 20°C after infection with CPMV did not develop infection symptoms. Symptoms of CPMV infection were more severe in plants incubated at 30°C compared to those incubated at 25°C, matching the higher CPMV titers in the apoplast under these conditions.

**Supplementary Movie 1: Simulation of microgravity using a custom-built random positioning machine (RPM).** Plants were mounted in the center of the device to minimize residual acceleration. The RPM was housed in a walk-in growth chamber to allow the control of temperature, humidity and photoperiod. In default experiments, a temperature of 25/22°C (day/night cycle), 60% relative humidity and ~100,000 lux (16-h photoperiod) were used. The light source was positioned outside of the RPM, thus preventing plants from orienting themselves based on the angle of light incidence.
